# An observational study of growth rate and body weight variance partition for United Kingdom dairy calves from birth to 20 weeks of age

**DOI:** 10.3168/jdsc.2020-0068

**Published:** 2021-05-21

**Authors:** S.C. Archer

**Affiliations:** School of Veterinary Medicine, University of Surrey, Guildford, Surrey GU2 7AL, United Kingdom

## Abstract

This study estimates the birth weight and growth rate for UK dairy calves and partitions unexplained variability in the weight of calves aged up to 20 wk. Raising replacements is a major contributor to the sustainability of dairying, but existing industry targets may not be applicable and achievable on all farms. For hierarchical data, variance partition coefficients (VPC) describe the proportion of unexplained variance residing at each level of the hierarchy due to clustering and indicate the potential to change outcomes through influencing variables acting at each level. This information could be used to inform the allocation of resources for further investigation. Calves were weighed according to farm-specific protocols. The data set analyzed contained records between June 5, 2014, and February 28, 2020, from 28 veterinary practices servicing 139 farms with 19,708 calves up to 20 wk of age, from which there were 59,588 weight recordings. Calves were Holstein-Friesian females. Calf weight was described using a multivariable mixed linear model with fixed effects for age. Mean birth weight was 41 kg. Mean growth rate increased from 0.59 kg/d at 1 d of age to 0.87 kg/d after 138 d. Cumulative mean growth rate up to 138 d of age was 0.73 kg/d. Birth weight and growth rate estimates are comparable with those made previously but are more precise due to larger sample size. Calf growth rate varied between farms, meaning that VPC at the calf, farm, and veterinary practice levels depended on calf age. Most unexplained variation in the weight of calves aged 66 d and over was due to differences between farms. At birth and 130 d of age, VPC at the farm level was 0.02 and 0.77, respectively. In contrast, most variation in neonatal calf weight was due to differences between calves. At birth and 130 d of age, VPC at the calf level was 0.84 and 0.20, respectively. The 0.025 to 0.975 quantile coverage of cluster-specific mean calf birth weight for combinations of veterinary practice and farm was 34 to 49 kg. The 0.025 to 0.975 quantile coverage of cluster-specific cumulative mean calf growth rate for combinations of veterinary practice and farm was 0.56 to 1.00 kg/d. Understanding reasons for these differences should be the basis of research into optimal calf management strategies to define economic targets for specific circumstances.

## Highlights

• In neonates, most variation in weight was due to differences between calves.
• From 66 d of age, most variation in weight was due to differences between farms.
• Farm-specific targets may be more useful than a “one size fits all” approach.

## Graphical Abstract

**Figure:**
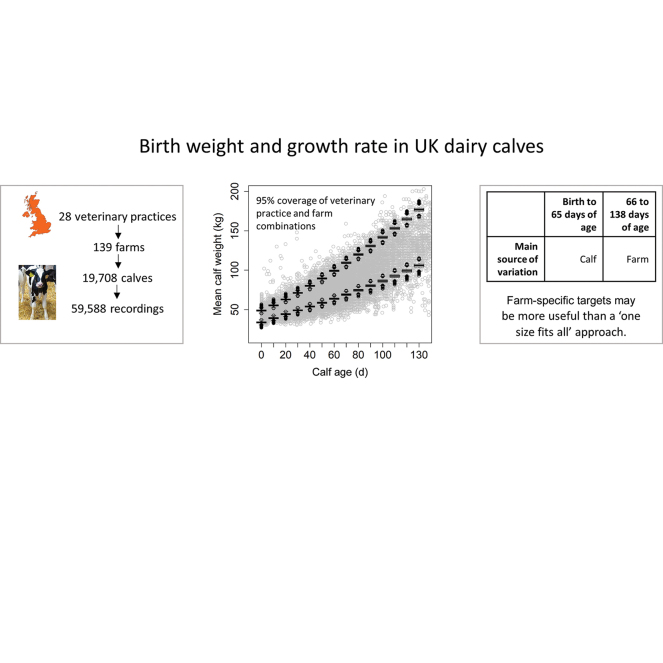
**Summary:** Sources of variation can be used to target resources to maximize potential intervention effect size. This research identified sources of variation in dairy calf birth weights and growth rates. Unexplained variance was partitioned proportionally to veterinary practice, farm, and calf levels (variance partition coefficient; VPC). At birth VPC was highest at the calf level, but this decreased with age. From 66 d of age onward, VPC was increasingly higher at the farm level. These trends are suggestive of the increasing influence of management and environment as calves age. This information can be used to inform strategies to investigate and optimize the efficiency of calf rearing systems.

Following a long history of research (Heinrichs et al., 2017), attention has been paid to monitoring the productivity of youngstock rearing operations on UK dairy farms (Bazeley et al., 2016). Increasing calf growth rate can potentially lead to gains in first-lactation milk yield (Soberon and Van Amburgh, 2013). Other motivating factors likely include trends for fewer, increasingly specialized dairy farms, availability of precision farming technologies, and recognition of the scale of economic losses (Tozer and Heinrichs, 2001).

Target growth rates are based on the mean calf (Brickell et al., 2009; AHDB Dairy, 2018), yet the mean calf does not exist, and extreme variability between farms and between calves within farms has been noted (Brickell et al., 2009). Such targets are unlikely to be relevant and achievable for all calves on all farms because of generalizability limitations and unexplained variation. Farm-specific target setting has been exemplified by the benchmarking approach (Atkinson et al., 2017). However, arithmetic means give a biased measure of central tendency, and an improvement would be to use targets based on proportions of calves within a given weight range for their age. Proportional targets cannot be robustly extrapolated from frequentist analyses but could be informed by the posterior predictive distributions obtained from a Bayesian approach (Spiegelhalter et al., 2004).

Statistical models offer explanations for some variation in complex data through fixed effects defined by what is known. Remaining residual variation is due to unknown variables or random noise. For hierarchical data such as calves clustered within farms, the variance partition coefficient (**VPC**) describes the proportion of unexplained variance residing at each level of the hierarchy due to clustering (Goldstein et al., 2002). This indicates potential change though influencing variables acting at each level. The same principle is routinely applied in dairy management, where genetic gain is leveraged through targeting characteristics with relatively large genetic variance components in selective breeding decisions (Macedo et al., 2021). Similarly, information on sources of variation could be used to target resources for investigating calf management to influence production efficiency.

The aim of this research was to update reported estimates of birth weight and growth rate for UK Holstein-Friesian female dairy calves and to partition unexplained variance in calf weight up to 20 wk of age. Reporting is based on Strengthening the Reporting of Observational Studies in Epidemiology–Veterinary Extension (STROBE) statement guidelines (Sargeant et al., 2016).

Since at least 2014, a subscription-based online service has been available to support the recording and monitoring of calf weight and health on a national scale in the UK (Calf Tracker, Zoetis). Veterinary practices have accounts to access the service, allowing upload of individual calf data from client farms. Collection and recording of calf identity and weights took place according to farm-specific protocols. Calf Tracker data from June 5, 2014, to February 28, 2020, were made available by Zoetis. The database contained records from 74 UK veterinary practices, with 526 farms classified as dairy, 45,736 calves, and 106,411 weight recordings. Calves were Holstein-Friesian females (David Bartram, Zoetis; personal communication). Likely due to errors in data entry, there were extreme and implausible values for calf age; however, 92,298 recordings from calves aged 1 to 138 d were deemed reliable and retained. To selectively recruit calves with repeated weight measurements, data were excluded from farms with ≤12 mo of apparent monitoring activity based on dates of first and last recordings in the database. Weight measurements denoted as estimates were removed. Confirmed birth weights for 19,715 calves were included as weight measurements for age 0 d, with recording date aligned to date of birth. Weight recordings were restricted to >30 and ≤225 kg. The selected data set contained records from 28 veterinary practices servicing 139 farms with 19,708 calves aged up to 138 d, from which there were 59,588 recordings (Table 1). For included calves, the median number of weight measurements was 3 (interquartile range: 2–3; range: 1–17).

**Table 1:** Inclusion counts (no.) of veterinary practices, farms, calves, and weight recordings by stages of data selection

Selection	Veterinary practices	Farms	Calves	Weight recordings
Recordings dated Jun. 5, 2014, to Feb. 28, 2020, from UK veterinary practices	74	526	45,736	106,411
Calves aged 1 to 139 d	73	498	41,046	92,298
Farms recording for >12 mo	28	142	26,348	60,913
Birth weights included as d 0 weight	28	139	19,715	59,854
Confirmed weights restricted to >30 kg and ≤225 kg	28	139	19,708	59,588

The data were initially explored using R (R Core Team, 2013). Subsequent modeling used R2MLwin (Zhang et al., 2016). The outcome of interest (*y_ijk_*) was the test-day weight of the *i*th calf (kg), on the *j*th farm, and from the *k*th veterinary practice. A mixed model was fitted to the data and took the form

$$\begin{array}{c} y_{ijk}=\alpha +{\text{X}}_{\text{ijk}}\cdot \beta +v_{k}+u_{jk}+e_{ijk},\\ \upsilon_{k}\sim N\left(0,\sigma_{\upsilon }^{2}\right),\\ u_{jk}\sim N\left(0,\sigma_{u}^{2}\right),\\ e_{ijk}\sim N\left(0,\sigma_{e}^{2}\right), \end{array}$$

where α = intercept value; **X**_ijk_ = a matrix of exposure variables for polynomials of calf age in days; **β** = a vector of coefficients for **X**_ijk_; *v_k_* = a random effect to adjust for clustering within veterinary practices, assumed to be normally distributed with mean 0 and variance $\sigma_{\upsilon }^{2};$ *u_jk_* = a random effect to adjust for clustering within farms, assumed to be normally distributed with mean 0 and variance $\sigma_{u}^{2};$ and *e_ijk_* = residual error, assumed to be normally distributed with mean 0 and variance $\sigma_{e}^{2}$. Calf age in days was forced into the model and investigated as polynomial terms. In this framework, **β** could be interpreted as a function of growth rate, and α could be interpreted as an estimate of birth weight. Month and year of measurement were investigated for inclusion as categorical terms. A plausible random slope for calf age was assessed for inclusion at the farm level. Parameters were initially estimated using iterative generalized least squares (Goldstein, 2003). Model fit was assessed by inspection of residuals for normality and by graphical comparison of predictions to raw data. To obtain robust credibility intervals for random effect variances, posterior predictive distributions for parameters were generated in a Bayesian framework (Zhang et al., 2016). Default noninformative prior distributions were used (Browne, 2015) for random effect variances as follows: $\sigma_{\upsilon }^{-2}\sim Gamma\left(0.001,0.001\right)$, $\sigma_{e}^{-2}\sim Gamma\left(0.001,0.001\right)$, and $\sigma_{u}^{-2}\sim {\text{Wishart}}_{2}\left(2{\text{S}}_{\text{u}},20\right);{\text{S}}_{\text{u}}=\left[\begin{array}{cc} 4.391 & \\ 0.024 & 0.017 \end{array}\right]$. Posterior predictive distributions for parameters were estimated from a sample of 10,000 Markov chain Monte Carlo simulations. To reduce autocorrelation, this was obtained by selecting every hundredth estimate in 1,000,000 Markov chain Monte Carlo simulations, following a burn-in of 100,000 simulations. Chain convergence was assessed by visual inspection to ensure that a stationary distribution had been reached and that chain length was consistent with estimates from the Rafferty-Lewis diagnostic applied to variance parameters with probability of 0.95 that the 0.025 to 0.975 quantile would be accurate within 0.005. Effective sample size was recorded as a measure of autocorrelation (Browne, 2015). The final model was confirmed using deviance information criteria, where low values indicate a more parsimonious model (Spiegelhalter et al., 2002).

Birth weight of the median calf on the median farm was 40 kg (interquartile range: 35–40 kg). At 20 wk of age, the median calf on the median farm was 136 kg (interquartile range: 117–159 kg). Between these time points, calf weight varied among calves, farms, and veterinary practices. Variability in estimates of calf weight increased with age, and sample size decreased as calves aged.

The final model included calf age as a quadratic term (Table 2). The intercept gives an estimate for mean birth weight (95% Bayesian credible interval, **BCI**) of 41 kg (BCI: 40–43 kg) after adjusting for missing data and clustering. Mean growth rate increased from 0.59 kg/d (BCI: 0.56–0.61 kg/d) at 1 d of age to 0.87 kg/d (BCI: 0.84–0.89 kg/d) at 138 d of age. Cumulative mean growth rate for included calves up to 138 d of age was 0.73 kg/d (BCI: 0.70–0.75 kg/d). Seasonal or temporal trends were not apparent. Month and year of measurement did not meaningfully influence the estimates of interest defined in the aim and were excluded from the final model.

**Table 2:** Final model for calf weight (kg) with estimates based 10,000 Markov chain Monte Carlo simulations using data from 28 veterinary practices, 139 farms, 19,708 calves, and 59,588 weight recordings

Model term	Fixed part: mean	Random part: variance	95% credible interval for mean or variance	Effective sample size
Intercept	41.26		39.86 to 42.67	1,417
Calf age (d)	0.59		0.56 to 0.61	2,008
Calf age^2^ (d^2^)	0.001		0.001 to 0.001	10,000
Vet practice (1)^1^		13.04	6.85 to 23.79	8,686
Farm (1)		2.37	1.65 to 3.34	10,429
Farm (covariance; 1, calf age)		0.007	−0.046 to 0.060	10,000
Farm (calf age)		0.019	0.014 to 0.024	10,000
Calf (1)		82.65	81.71 to 83.57	10,491

1 Intercept.

The final model included a random slope for calf age at the farm level. Covariance between birth weight and growth rate was positive in 66% of simulations (Table 2), leading to a fanning pattern in growth curves (see graphical abstract). Variance partition coefficient estimates depended on calf age (Figure 1). At the farm level, mean VPC increased from 0.02 (BCI: 0.02–0.03) for calves aged 0 d to 0.77 (BCI: 0.72–0.81) for calves aged 130 d. Most variation was at the farm level in more than half of simulations for calves aged 66 d and older. At the veterinary practice and calf levels, there were trends for decreasing VPC with increasing calf age. At birth VPC was highest at the calf level (0.84; BCI: 0.76–0.90) but declined to 0.20 (BCI: 0.17–0.24) at 130 d.

**Figure 1:**
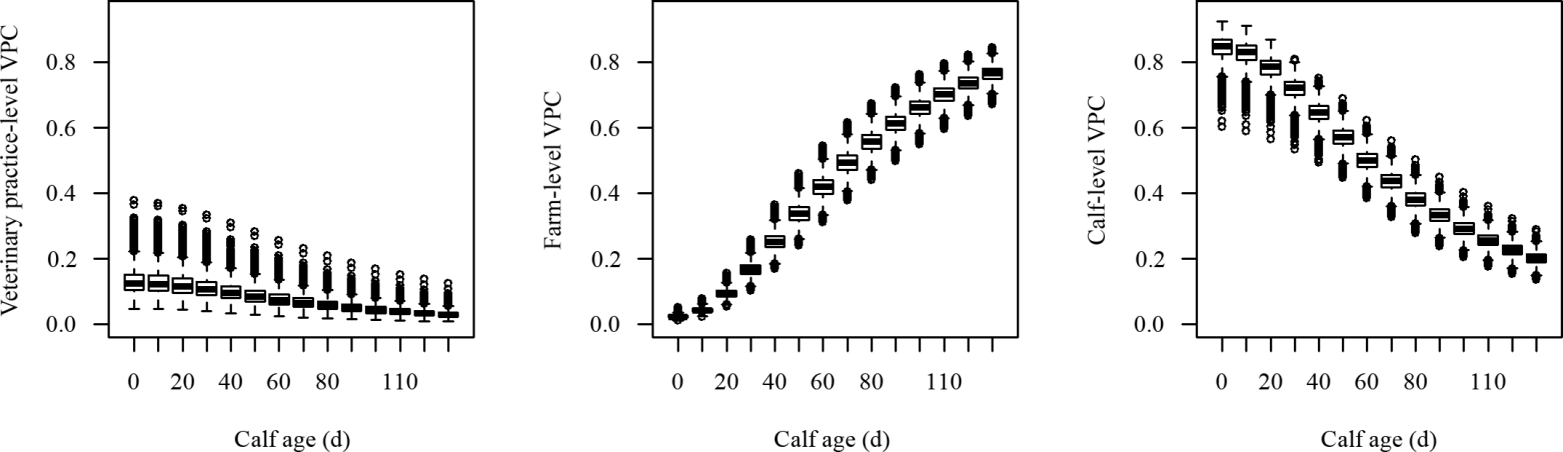
Final model predictions of variance partition coefficients (VPC) at the veterinary practice, farm, and calf levels. The final model included a random slope for calf age at the farm level. The boxplots summarize predictions from 10,000 Markov chain Monte Carlo simulations of VPC for calves aged 0 to 130 d (bold horizontal lines are the median, the box is the interquartile range, the whiskers span the quantile from 0.025 to 0.975, and the circles are outliers).

The 0.025 to 0.975 quantile coverage of cluster-specific mean calf birth weight for combinations of veterinary practice and farm was 34 kg (BCI: 31–36 kg) to 49 kg (BCI: 47–52 kg). The 0.025 to 0.975 quantile coverage of cluster-specific cumulative mean calf growth rate up to 138 d of age for combinations of veterinary practice and farm was 0.56 kg/d (BCI: 0.52–0.60 kg/d) to 1.00 kg/d (BCI: 0.96–1.04 kg/d), respectively.

This is the largest reported study of repeated weight measurements in dairy calves from birth to beyond weaning and the first study to partition variance in age-adjusted calf weights. Central tendency measures of birth weight and cumulative mean growth rate were consistent with previous reports (Brickell et al., 2009; Bazeley et al., 2016). In these studies, birth weight and growth rate in dairy calves have been estimated from smaller samples of UK farms and calves, yet variability in these parameters has been a notable finding (Brickell et al., 2009; Bazeley et al., 2016). Previously, the largest study was based on 5,273 Holstein heifers from 163 farms in Pennsylvania (Heinrichs and Hargrove, 1987), and in the United Kingdom a study of 509 calves on 19 dairy farms has informed industry recommendations (Brickell et al., 2009; AHDB Dairy, 2018). Brickell et al. (2009) estimated a cumulative mean daily growth rate of 0.77 kg/d (range: 0.45–1.13 kg/d) in calves up to 6 mo of age. Comparable mean daily growth rate on the median of 8 dairy farms over at least 730 d was 0.71 kg/d (range: 0.58–0.75 kg/d; Bazeley et al., 2016). On these 8 farms the relationship between calf weight and age also appeared to be quadratic, with growth rate progressively increasing with time from 0.29 kg/d (range: 0.00–0.52 kg/d) up to 30 d of age (Bazeley et al., 2016). Six of the 8 dairy farms had 348 calves with mean and median birth weight of 40 kg (range: 24–55 kg; Bazeley et al., 2016).

Variability in calf birth weight and growth rate estimates can be expected to decrease with increasing sample size. The current study therefore produced more precise estimates of mean birth weight (41 kg; BCI: 40–43 kg) and cumulative mean growth rate (0.73 kg/d; BCI: 0.70–0.75 kg/d), although different ages of calves were considered. The modeling approach adjusts for lack of independence between calves, meaning that estimates of central tendency apply to calves on the median farm serviced by the median veterinary practice. Coverage of cluster-specific means shows diversity between farms and veterinary practices. For the bottom 2.5% of farms serviced by the bottom 2.5% of veterinary practices, estimates of mean calf birth weight and cumulative mean growth rate were ≤34 kg (BCI: 31–36 kg) and ≤0.56 kg/d (BCI: 0.52–0.60 kg/d), respectively. For the top 2.5% of farms serviced by the top 2.5% of veterinary practices, estimates of mean calf birth weight and cumulative mean growth rate were ≥49 kg (BCI: 47–52 kg) and ≥1.00 kg/d (BCI: 0.96–1.04 kg/d), respectively. The 95% BCI reported are based on posterior predictive distributions of parameters, meaning that they can be applied directly as probability statements to inform decision making (Spiegelhalter et al., 2004) and used to develop farm-specific targets. For example, setting targets at the BCI lower bound would give a minimum target applicable to 97.5% of calves from a given percentile of farms.

A large change in cumulative mean growth rate (equivalent to a farm in the bottom 2.5% moving to the top 2.5%) would require one or more interventions to increase cumulative calf growth rate by ≥0.44 kg/d. Such a change may be associated with an increase in mean first-lactation milk yield of 682 ± 280 kg (Soberon and Van Amburgh, 2013). The marginal value of any additional milk would be indicative of the potential intervention budget. Further research is needed to inform farm-specific budgets for appropriate interventions to increase the chance that changes are cost effective.

Mean calf growth rate varied between farms, such that sources of unexplained variation in weight were dependent on calf age. Influential farm-level differences could have been related to milk or colostrum feeding and environmental conditions in terms of hygiene and thermal comfort (Hyde et al., 2021). However, for neonatal calves, most unexplained variation in calf weight was at the calf level, relating to differences between calves within farms (Figure 1). This could indicate variation in the application of management or environmental conditions over time. Missing explanatory variables could relate to genetic differences between calves, differences in dam characteristics such as parity, or differences in the viability of individual calves due to the effectiveness of passive transfer of immunity from colostrum, nutrition, and diseases such as diarrhea and pneumonia (Windeyer et al., 2014). Commercial information known to the database owners showed that calves were Holstein-Friesian females. It is possible that this assumption was unreliable in some cases, although favorable comparison of central tendencies for birth weights and growth rates with prior expectations suggests that any bias due to misclassification of breed and sex was inconsequential.

Compared with unknown variables acting at the calf level, any acting at the veterinary practice level were of lesser importance. This indicates that characteristics that could distinguish veterinary service providers are of limited importance in terms of calf birth weight and that the relative importance declines further as calves age. From 66 d of age, unexplained variation at the farm level exceeded that at the calf level. This could be due to increasing exposure to common environmental and management factors within farms that diminish the relative importance of phenotypic differences between calves over time. The development of optimal calf management strategies that realize genetic potential could be informed by research to identify reasons for differences in calf birth weight and growth rates among calves, farms, and veterinary practices throughout the rearing period. Results shown in Figure 1 can be applied to inform the allocation of resources for investigations. Studies in calves <66 d of age should prioritize factors underlying calf-level differences, such as differences in genotype, disease status, or failure of passive transfer. Studies in calves ≥66 d of age should prioritize factors underlying farm-level differences, such as differences in environment and management.

Results of this research are conditional on calves being eligible for weighing through survival. This could give an overly optimistic representation of calf production on the selected farms by being based on the best calves. Reasons for decreasing numbers of calves weighed with increasing age are unknown; however, calf mortality as a contributory factor (Brickell and Wathes, 2011; Hyde et al., 2020) should be considered before developing calf management strategies based on weight measurements alone.

Optimal calf-rearing strategies should aim to maximize lifetime productivity for the least cost. Heinrichs et al. (2017) reviewed a large body of research focused on developing growth standards for dairy heifers, ranging from small uncontrolled experiments to population studies. Accelerated growth during the rearing period to reduce costs has had mixed results in terms of first-lactation milk yield. High growth rates in the prepubertal period adversely influenced mammary development in some studies (Meyer et al., 2006) and were optimal at 0.80 kg/d in a meta-analysis of first-lactation milk yield (Zanton and Heinrichs, 2005). More than 99% of calves in this research remained <200 kg in weight, a value suggested to be an upper limit of the prepubertal period in Holsteins (Macdonald et al., 2005). Growth rates for calves on the median farm serviced by the median veterinary practice in this research approach this guideline. This ignores random effects due to farm, which have an increasing effect on calf growth rate as calves age. As growth rates varied between farms, there are opportunities to learn from a range of existing practices to optimize calf productivity.

This study was based on calves in a convenience sample of veterinary practices and farms. External validity has not been assessed. As such, mean birth weight and growth rate estimates should be extrapolated to the target population of UK dairy heifer calves with caution. This bias could lead to underestimation of unexplained variance, potentially increasing the relevance of this study.

In conclusion, mean birth weight and growth rate estimates were consistent with expectations. However, estimates varied substantially when random effects due to clustering of calves within farms and farms within veterinary practices were considered. In neonatal calves, most unexplained variance in weight was attributed to differences between calves within farms. Calf growth rate varied between farms, and difference between farms was the largest determinant of unexplained variance in calf weight from 66 d of age. Understanding reasons for these differences should be the basis of research into optimal calf management strategies.

## References

[bib1] AHDB Dairy (2018). Calf management. https://dairy.ahdb.org.uk/resources-library/technical-information/health-welfare/calf-management-factsheets/#.Xs-VyfZFy3A.

[bib2] Atkinson D.J., von Keyserlingk M.A.G., Weary D.M. (2017). Benchmarking passive transfer of immunity and growth in dairy calves. J. Dairy Sci..

[bib3] Bazeley K.J., Barrett D.C., Williams P.D., Reyher K.K. (2016). Measuring the growth rate of UK dairy heifers to improve future productivity. Vet. J..

[bib4] Brickell J.S., McGowan M.M., Wathes D.C. (2009). Effect of management factors and blood metabolites during the rearing period on growth in dairy heifers on UK farms. Domest. Anim. Endocrinol..

[bib5] Brickell J.S., Wathes D.C. (2011). A descriptive study of the survival of Holstein-Friesian heifers through to third calving on English dairy farms. J. Dairy Sci..

[bib6] Browne W.J. (2015). MCMC Estimation in MlwiN. Version 2.32. http://www.bris.ac.uk/cmm/media/software/mlwin/downloads/manuals/2-32/mcmc-web.pdf.

[bib7] Goldstein H. (2003).

[bib8] Goldstein H., Browne W., Rasbash J. (2002). Partitioning variation in multilevel models. Underst. Stat..

[bib9] Heinrichs A.J., Hargrove G.L. (1987). Standards of weight and height for Holstein heifers. J. Dairy Sci..

[bib10] Heinrichs A.J., Zanton G.I., Lascano G.J., Jones C.M. (2017). A 100-year review: A century of dairy heifer research. J. Dairy Sci..

[bib11] Hyde R.M., Green M.J., Hudson C., Down P.M. (2021). Factors associated with daily weight gain in preweaned calves on dairy farms. Prev. Vet. Med..

[bib12] Hyde R.M., Green M.J., Sherwin V.E., Hudson C., Gibbons J., Forshaw T., Vickers M., Down P.M. (2020). Quantitative analysis of calf mortality in Great Britain. J. Dairy Sci..

[bib13] Macdonald K.A., Penno J.W., Bryant A.M., Roche J.R. (2005). Effect of feeding level pre- and post-puberty and body weight at first calving on growth, milk production, and fertility in grazing dairy cows. J. Dairy Sci..

[bib14] Macedo F.L., Christensen O.F., Legarra A. (2021). Selection and drift reduce genetic variation for milk yield in Manech Tête Rousse dairy sheep. JDS Commun..

[bib15] Meyer M.J., Capuco A.V., Ross D.A., Lintault L.M., Van Amburgh M.E. (2006). Developmental and nutritional regulation of the prepubertal heifer mammary gland: I. Parenchyma and fat pad mass and composition. J. Dairy Sci..

[bib16] R Core Team (2013). A Language for Statistical Computing. http://www.r-project.org/.

[bib17] Sargeant J.M., O'Connor A.M., Dohoo I.R., Erb H.N., Cevallos M., Egger M., Ersbøll A.K., Martin S.W., Nielsen L.R., Pearl D.L., Pfeiffer D.U., Sanchez J., Torrence M.E., Vigre H., Waldner C., Ward M.P. (2016). Methods and processes of developing the Strengthening the Reporting of Observational Studies in Epidemiology – Veterinary (STROBE-Vet) statement. J. Vet. Intern. Med..

[bib18] Soberon F., Van Amburgh M.E. (2013). Lactation biology symposium: The effect of nutrient intake from milk or milk replacer of preweaned dairy calves on lactation milk yield as adults: A meta-analysis of current data. J. Anim. Sci..

[bib19] Spiegelhalter D., Abrams K., Myles J. (2004).

[bib20] Spiegelhalter D., Best N., Carlin B., van der Linde A. (2002). Bayesian measures of model complexity and fit. J. R. Stat. Soc. B.

[bib21] Tozer P.R., Heinrichs A.J. (2001). What affects the costs of raising replacement dairy heifers: A multiple-component analysis. J. Dairy Sci..

[bib22] Windeyer M.C., Leslie K.E., Godden S.M., Hodgins D.C., Lissemore K.D., LeBlanc S.J. (2014). Factors associated with morbidity, mortality, and growth of dairy heifer calves up to 3 months of age. Prev. Vet. Med..

[bib23] Zanton G.I., Heinrichs A.J. (2005). Meta-analysis to assess effect of prepubertal average daily gain of Holstein heifers on first-lactation production. J. Dairy Sci..

[bib24] Zhang Z., Parker R.M.A., Charlton C.M.J., Leckie G., Browne W.J. (2016). R2MLwiN: A package to run MLwiN from within R 72. J. Stat. Softw..

